# Immunometabolic editing of the tumor microenvironment: from reprogramming mechanisms to therapeutic vulnerabilities

**DOI:** 10.3389/fimmu.2025.1678446

**Published:** 2026-01-16

**Authors:** Yixin Li, Yunmei Zhang, Yan Nie, Xueman Chen

**Affiliations:** 1 Guangdong Provincial Key Laboratory of Malignant Tumor Epigenetics and Gene Regulation, Breast Tumor Center, Sun Yat-Sen Memorial Hospital, Sun Yat-Sen University, Guangzhou, China; 2 Guangdong-Hong Kong Joint Laboratory for RNA Medicine, Breast Tumor Center, Sun Yat-Sen Memorial Hospital, Sun Yat-Sen University, Guangzhou, China; 3 Guangdong Provincial Key Laboratory of Malignant Tumor Epigenetics and Gene Regulation, Medical Research Center, Sun Yat-sen Memorial Hospital, Sun Yat-sen University, Guangzhou, China; 4 Guangdong-Hong Kong Joint Laboratory for RNA Medicine, Medical Research Center, Sun Yat-sen Memorial Hospital, Sun Yat-sen University, Guangzhou, China; 5 Guangdong Provincial Key Laboratory of Cancer Pathogenesis and Precision Diagnosis and Treatment, Sun Yat-sen Memorial Hospital, Sun Yat-sen University, Shanwei, China

**Keywords:** immune cells, metabolic reprogramming, stromal cells, tumor cells, tumor immunometabolism, tumor immunotherapy, tumour microenvironment (TME)

## Abstract

Metabolic reprogramming is not only one of the malignant characteristics of tumor cells, but also commonly seen in a variety of immune cells in tumor microenvironment(TME), which massively promotes tumor-body immune interaction. Immunometabolic editing is a dynamic, co-evolutionary process wherein adaptive metabolic reprogramming in the TME, driven by tumor-immune crosstalk during immunoediting, critically shapes anti-tumor immune response and governs immune evasion. Studies of metabolic pathways linked to anti-tumor immune process and discoveries of important therapeutic targets are conducive to the development of targeted immunometabolic intervention to enhance the body’s anti-tumor immune response and improve the efficacy of tumor immunotherapies. This review summarizes metabolic characteristics of the TME, highlights immunometabolic editing during cancer evolution, and discusses mechanisms by which tumor immunotherapies modulate tumor immunometabolism to identify potential therapeutic targets.

## 1 Introduction

Metabolic reprogramming is a hallmark of cancer, where tumor-induced alterations in metabolic pathways regulate diverse immune cell functions to achieve immunosuppression. Specifically, tumor cells rewire metabolic circuits (e.g., glycolysis, glutaminolysis) to compete for nutrients in the tumor microenvironment (TME), thereby impairing effector T cell function while promoting immunosuppressive populations like myeloid-derived suppressor cells (MDSCs) and regulatory T cells (Tregs) (1, 2). The metabolic interaction between tumor cells and immune cells and how these cells adapt to the TME by forming corresponding metabolic phenotypes are the key factors that determine tumor immune response, immune escape or immunotherapy. From an evolutionary and integrative perspective, we here propose the concept of Immunometabolic Editing—a dynamic and reciprocal process in the TME wherein metabolic competition and crosstalk throughout the immunoediting cascade drive the selection of tumor variants with specific metabolic profiles, while simultaneously shaping the functional fate of immune infiltrates. This co-evolution ultimately determines immunological control versus tumor escape.

Therefore, targeting key nodes in tumor immunometabolism and synergizing with existing immunotherapies has emerged as a promising frontier to overcome resistance and enhance therapeutic efficacy. By addressing the metabolic demands of immune cells (3), such strategies synergistically enhance immunotherapy efficacy (4). This integrated approach offers novel therapeutic avenues to overcome metabolic immunosuppression and potentiate antitumor immune responses, as evidenced by preclinical and clinical advances in metabolic modulators and nanomedicine-based delivery systems (5).

In this review, we will first give an overview of metabolic properties in tumor microenvironment. We will also highlight the process of immunometabolic editing, presenting metabolic alterations of tumor cells, immune cells, as well as stromal cells in the TME, and summarize how they support cancer evolution. Last but not least, we will illustrate mechanisms of tumor immunotherapies underlying tumor cell metabolism regulation, technological frontiers in dissecting tumor immunometabolism, and propose promising therapeutic targets in tumor immunometabolism.

## 2 Metabolic properties in tumor microenvironment

The metabolic interplays between tumor cells and stromal components within the TME exist throughout the entire process of tumor evolution. Tumor cells exhibit a distinct metabolic phenotype characterized by aerobic glycolysis (Warburg effect), even under normoxic conditions, to prioritize biomass synthesis over ATP efficiency. This metabolic shift supports rapid cell proliferation by generating glycolytic intermediates for nucleotides, amino acids, and lipids (6). Enhanced lactate dehydrogenase (LDH) activity in tumor cells further drives lactate production, acidifying the TME and promoting immunosuppression (7). Stromal cells, particularly cancer-associated fibroblasts (CAFs), adopt a “reverse Warburg effect”, engaging in aerobic glycolysis to secrete lactate, pyruvate, and glutamine, which would be exploited by tumor cells for oxidative phosphorylation and anabolic pathways (8–10). This metabolic symbiosis sustains tumor growth while depleting glucose reserves, creating a nutrient-deprived TME (11).

These metabolic interplays also have an impact on immunoactivity. First of all, tumor cells’ hyperactive glycolysis exhausts extracellular glucose, impairing effector T cell functions and fostering immune evasion. Glucose deprivation in tumor-infiltrating lymphocytes (TILs) suppresses mTOR signaling, diminishes glycolytic flux, and impairs interferon-gamma (IFN-γ) transcriptional output, culminating in a progressive impairment of immune effector functions (12). Elevated lactate (10–40 mM) acidifies the TME (pH ~6.5–6.9), inhibiting cytotoxic T lymphocyte (CTL) activity and promoting lipid synthesis via SLC5A12-mediated uptake in stromal cells (13, 14). Also, the elevated extra-cellular lactate levels within the TME may impair the efflux capacity of glycolytically active dendritic cells, thereby contributing to progressive intracellular lactate retention (15). On the other hand, low oxygen gradients within the TME activate HIF-1α, which upregulates glycolytic enzymes like glucose transporters (GLUT1) or hexokinase 2 (HK2), and PDK1 to suppress mitochondrial pyruvate utilization, redirecting carbon flux toward lactate (16). Hypoxia also induces lipid droplet formation in tumor-associated macrophages (TAMs), driving M2 polarization and immune suppression (17). Tumor cells and stromal cells exhibit increased lipid storage via upregulated enzymes of *de novo* lipogenesis and lipid scavenging. Lipid-rich TAMs adopt pro-tumor phenotypes through PI3K-γ/STAT6 signaling, while lipid droplets in cancer cells enhance chemoresistance and metastasis (18–20). These metabolic adaptations create a feed-forward loop: hypoxia and acidosis reinforce glycolytic flux, lactate sustains lipid biosynthesis, and lipid-laden stromal cells further compromise antitumor immunity. Targeting these pathways—e.g., inhibiting HIF-1α, LDHA, or fatty acid oxidation—may disrupt TME-driven immunosuppression and improve therapeutic outcomes (21, 22). More details of metabolic properties in the TME are demonstrated in **Table 1**.

**Table 1:** An overview of metabolic reprogramming of tumor cells.

Category	Characteristics	Mechanisms	Results
Glucose Metabolism	Enhanced Aerobic Glycolysis	Tumor cells preferentially utilize glycolysis for ATP generation.	Excessive glucose consumption and lactate production
	Lactate Accumulation and Acidification	High glycolytic flux leads to lactate secretion into the TME via MCTs.	The polarization of TAMs towards an immunosuppressive phenotype.
	Competition for Glucose	Highly glycolytic tumor cells deplete local glucose, creating nutrient restriction.	Impairs the function of glucose-dependent anti-tumor immune cells, leading to their dysfunction or exhaustion.
	Metabolic Coupling with CAFs	CAFs exhibit increased glycolytic activity, secreting pyruvate and lactate	Utilized by adjacent tumor cells.
	Hypoxia-Induced Glycolysis	Tumor-associated hypoxia, via HIF-1α stabilization	Amplifies glycolysis and lactate production
	Key Enzyme Dysregulation	Enzymes like HK2, PKM2, and LDH are overexpressed or dysregulated in the TME	Drive glycolytic flux and contribute to immune suppression
Amino Acid Metabolism	Glutamine Addiction	Tumor cells exhibit increased glutaminolysis.	Impairs nucleotide synthesis, TCA cycle anaplerosis, glutathione synthesis.
	Tryptophan Depletion and Kynurenine Production	Tumor and myeloid cells overexpress indoleamine or dioxygenase, depleting tryptophan and producing immunosuppressive kynurenine metabolites.	Inhibits effector T-cell function, promotes regulatory T-cell induction.
	Arginine Metabolism Dysregulation	M1 Macrophages: Utilize iNOS to convert arginine into NO M2 TAMs & Myeloid Cells: Express high levels of ARG1, depleting arginine and producing ornithine and polyamines.	Inhibits T-cell proliferation and function, while polyamines support tumor growth.
	Cysteine/Methionine Dependency	Tumor cells often rely on uptake of cysteine and methionine for glutathione synthesis, protein translation, and methylation reactions.	Limits availability for immune cells.
	Serine/Glycine Biosynthesis	*De novo* serine and glycine synthesis pathways are enhanced	Support nucleotide synthesis and one-carbon metabolism crucial for rapid proliferation
	Metabolic Crosstalk with CAFs	CAFs can secrete amino acids to support tumor cell proliferation and invasion.	ECM remodeling and invasion.
Lipid Metabolism	Increased Lipid Uptake and Storage	Tumor cells upregulate fatty acid transporters to import FFAs released from adipocytes or CAFs via lipolysis.	FFAs are stored as LDs for energy reserves or membrane synthesis.
	Enhanced *De Novo* Lipogenesis	Despite nutrient availability, many tumors upregulate key enzymes to synthesize fatty acids *de novo* from acetyl-CoA	Membrane biogenesis for rapid proliferation and providing signaling molecules.
	Fatty Acid Oxidation Adaptation	Some tumor subpopulations and specific TME cells increase FAO, particularly under hypoxia or nutrient stress	As an alternative energy source. CPT1A is a key regulator
	Cholesterol Metabolism Dysregulation	Tumor cells exhibit altered cholesterol uptake, synthesis, and efflux.	Act as signaling molecules, promoting tumor progression and modulating the immune response.
	Adipocyte Remodeling	Adipocytes adjacent to tumors undergo lipolysis, releasing FFAs and adipokines.	Promote ECM remodeling, inflammation, estrogen production, and tumor invasion/metastasis.
	CAF-Mediated Lipid Support	CAFs can undergo lipolysis and release FFAs. They also secrete lipid mediators.	Fuel adjacent tumor cells

MCTs, monocarboxylate transporters; HK2, Hexokinase 2; PKM2, pyruvate kinase M2; LDH, lactate dehydrogenase; iNOS, inducible nitric oxide synthase; NO, nitric oxide; ARG1, Arginase 1; CAFs, cancer-associated fibroblasts; ECM, extracellular matrix; FFAs, free fatty acids; LDs, lipid droplets; FAO, fatty acid oxidation; CPT1A, carnitine palmitoyl transferase 1A; FFAs, free fatty acids.

## 3 Immunometabolic editing during cancer evolution

### 3.1 Metabolic reprogramming in tumor immune response

**Figure 1:**
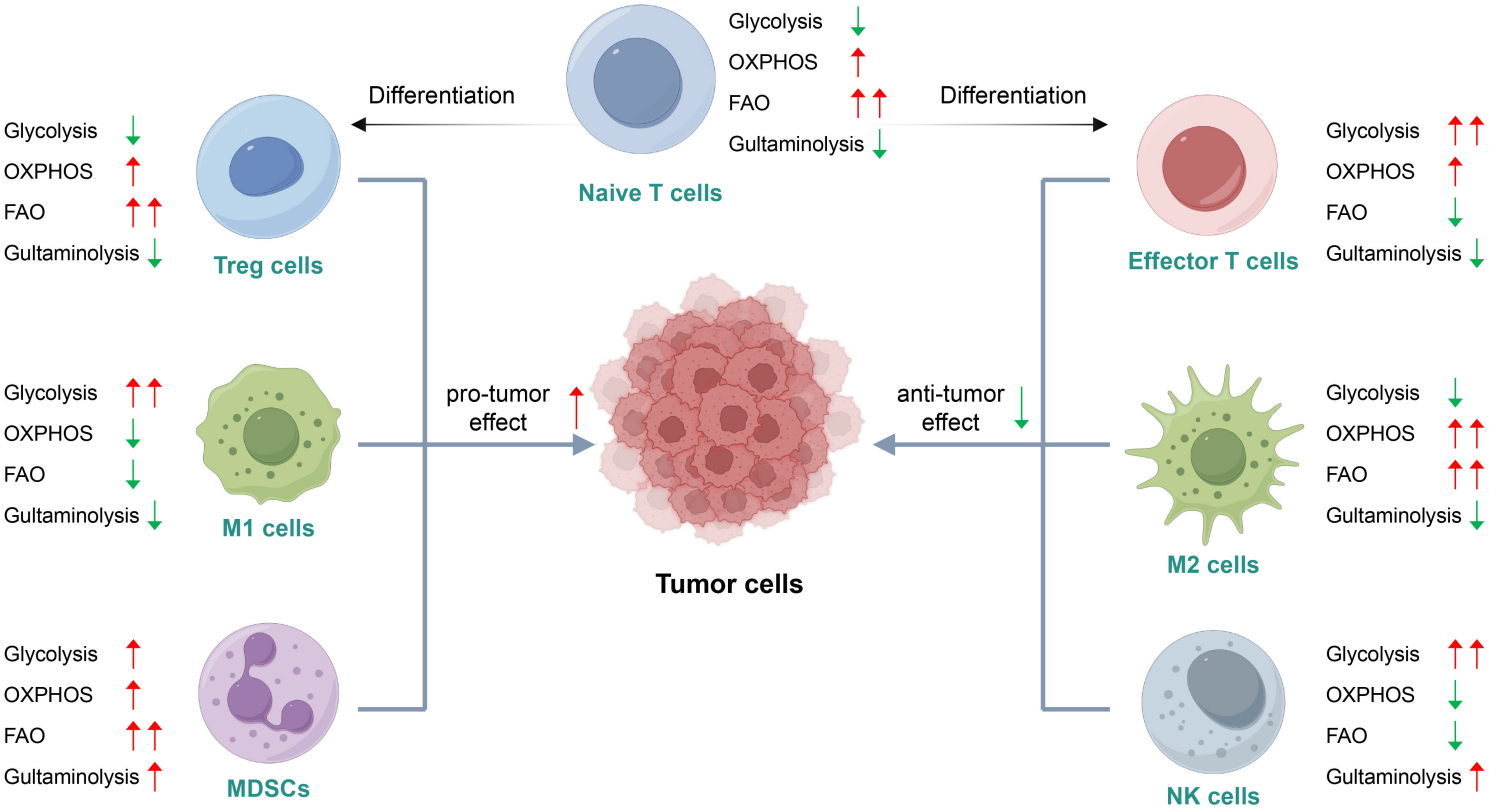
Metabolism of immune cells is distinguishably reprogrammed in the TME. The reprogrammed metabolism of immune cells exhibits stage-specific divergence between activation states and differentiation phases, with preferential engagement of distinct pathways to fulfill diverse functional demands. Conversely, systemic metabolic conditions and microenvironmental metabolites exert significant influence on immune cell phenotypes and effector functions. (Created with figdraw.com).

The metabolic interplay between tumor cells and immune cells during antitumor immune responses profoundly shapes immunoediting process and therapeutic efficacy. Effector immune cells exert anti-tumor activity and consume nutrients such as glucose and arginine, creating localized metabolic stress that can directly eliminate tumor cells or inhibit their growth, herein referred to as metabolic elimination. Recent advances highlight that immune cell metabolic reprogramming is dynamically regulated by activation states and TME constraints, exhibiting distinct patterns across immune subsets (23, 24) The overview of metabolic reprogramming is illustrated in **Figure 1**.

Naïve T cells rely on oxidative phosphorylation (OXPHOS) for energy, but upon activation, effector T cells undergo metabolic reprogramming toward aerobic glycolysis and glutaminolysis to meet biosynthetic demands for proliferation and cytokine production (23, 24). However, in the TME, tumor cells outcompete T cells for glucose via upregulated GLUT1, leading to CD8^+^ T cell exhaustion and impaired glycolytic flux (23, 25). Recent studies reveal that enhancing mitochondrial metabolism (e.g., via PGC-1α) or targeting adenosine/PD-L1 pathways can reinvigorate T cell function (26). Additionally, glutamine restriction in the TME disrupts T cell differentiation, but pharmacologic inhibition of tumor glutaminase (GLS1) enhances CTL infiltration and anti-tumor responses (27).

NK cells depend on glycolysis and OXPHOS for cytotoxicity. In hypoxic TME, mitochondrial dysfunction impairs NK cell effector functions, while lactate acidosis downregulates NKG2D receptor expression, reducing tumor cell recognition (28). Recent studies propose metabolic interventions such as LDH inhibitors to mitigate lactate toxicity or ROS scavengers like NAC to restore mitochondrial integrity, thereby enhancing NK-mediated tumor lysis (26). Notably, tissue-resident NK subsets exhibit unique metabolic dependencies on fatty acid oxidation (FAO), suggesting context-specific targeting opportunities (29).

Neutrophils undergo dynamic metabolic reprogramming during tumor immune surveillance, shifting between anti-tumor (N1) and pro-tumor (N2) phenotypes (30). Activated neutrophils primarily utilize glycolysis for ATP production and NETosis, facilitated by HIF-1α under hypoxia (31). This supports ROS generation via PPP for cytotoxic functions (32). Tumor-associated neutrophils (TANs) exhibit enhanced OXPHOS and FAO, particularly in immunosuppressive N2 states. This metabolic shift supports survival in nutrient-depleted TME and facilitates arginase-1-mediated T-cell suppression (32).

Macrophage polarization (M1/M2) is tightly linked to metabolic reprogramming. Pro-inflammatory M1 macrophages utilize glycolysis and broken TCA cycles, accumulating succinate to stabilize HIF-1α and drive IL-1β production. In contrast, anti-inflammatory M2 macrophages depend on OXPHOS and FAO, supported by enhanced glutamine metabolism and α-ketoglutarate (α-KG)-dependent epigenetic remodeling (33).

By redefining the metabolic landscape of the TME, immune cells either drive anti-tumor elimination or succumb to suppression, implying metabolic reprogramming as a critical axis for therapeutic intervention in overcoming immune evasion.

### 3.2 Metabolic reprogramming in tumor immune evasion

Immune pressure selects for tumor cell clones that has evolved superior metabolic fitness to survive in a metabolically hostile landscape or that can impose immunosuppressive metabolic constraints on immune cells, leading to metabolism-dominant immune escape. Emerging evidence highlights that tumor-derived metabolites and nutrient competition synergistically impair effector immune cell function while promoting immunosuppressive cell infiltration, thereby facilitating immune evasion during tumor development.

Tumor cells generate immunosuppressive metabolites through dysregulated glycolysis and amino acid metabolism. Lactate accumulation directly inhibits CTL proliferation and IFN-γ production (27). Lactate not only acidifies the TME but also directly suppresses NK-cell cytotoxicity and dendritic cell (DC) maturation, while enhancing Treg suppressive activity via FOXP3 and monocarboxylate transporter-1 (MCT-1) up-regulation (34). Glutamine deprivation in bladder cancer triggers EGFR/MEK/ERK/c-Jun signaling, elevating PD-L1 expression and impairing T-cell responses (35). Conversely, pharmacologic inhibition of glutaminase (e.g., JHU083) reprograms T-cell metabolism toward OXPHOS, enhancing antitumor immunity in preclinical models (36). Notably, tumor-derived kynurenine—a product of tryptophan catabolism via indoleamine 2,3-dioxygenase (IDO)—induces Treg differentiation while suppressing CD8^+^ T cell effector functions, creating a tolerogenic niche (29, 30).

Glucose and glutamine depletion in the TME starve effector T cells, triggers T cell exhaustion, characterized by upregulated PD-1 and CTLA-4 expression, which synergizes with PD-L1 overexpression on tumor cells to amplify immune checkpoint signaling (32, 33). Glucose deprivation impairs T-cell function by suppressing the mTOR signaling pathway. Limited glucose availability inhibits mTORC1, a crucial regulator of T-cell differentiation and effector functions (37). This suppression curtails T-cell proliferation and cytokine production, thereby weakening the anti-tumor immune response (38). Separately, glutamine deprivation can promote immune evasion by upregulating PD-L1 expression. This process is mediated through the activation of the EGFR/MEK/ERK/c-Jun signaling cascade (39, 40). The transcription factor c-Jun, activated downstream of EGFR and ERK, directly binds to the PD-L1 promoter, enhancing its expression and thus facilitating tumor escape from immune surveillance (41, 42). Recent studies reveal that mitochondrial dysfunction in CD8^+^ T cells—driven by tumor-induced oxidative stress—exacerbates metabolic insufficiency, impairing OXPHOS and perpetuating exhaustion (37, 38). Chronic antigen exposure and hypoxic stress in the TME induce mitochondrial fragmentation and bioenergetic failure in T cells. Exhausted T cells exhibit depolarized mitochondria, reduced OXPHOS, and impaired FAO, which are critical for sustaining effector functions (34). Furthermore, tumor-derived extracellular vesicles containing mitochondrial DNA (mtDNA) can paradoxically foster immune tolerance by activating the cGAS-STING pathway in stromal cells, increasing PD-L1 expression (43).

Concurrently, metabolite gradients actively reshape the immune landscape. Lactate and adenosine promote M2 macrophage polarization and Treg expansion via HIF-1α stabilization, while hypoxic regions secrete chemokines like CCL22 or CXCL12 to attract MDSCs (39, 40). These cells further deplete arginine and cysteine, metabolites essential for T cell receptor signaling and glutathione synthesis, respectively (41). Notably, tumor cholesterol esterification generates lipid rafts enriched in PD-L1, simultaneously enhancing immune checkpoint expression and recruiting lipid-dependent Tregs (31).

This metabolic reprogramming extends across the immune repertoire. While Teff and NK cells experience suppressed cytotoxicity and metabolic flexibility due to lactate accumulation, microenvironmental acidosis, and nutrient deprivation (44), suppressive populations like Tregs and MDSCs thrive on FAO and OXPHOS, leading to their expansion (34). In premetastatic niches, neutrophils utilize mitochondrial fatty acid metabolism to overcome glucose limitations, promoting immunosuppressive ROS production and disrupting nuclear factor of activated T cells (NFAT) signaling (34). Additional mechanisms include mitochondrial ROS accumulation in TAMs, which promotes M2 polarization and reinforces immunosuppression (36), along with AMPK signaling inhibition in γδ T cells due to a dominant Warburg effect, leading to impaired anti-tumor activity (35).

Metabolic-immune crosstalk also varies across malignancies. In melanoma, intratumoral heterogeneity correlates with reduced immune cytolytic activity and resistance to checkpoint inhibitors, partly due to metabolic subclones evading immune surveillance (45). Non-small cell lung cancers (NSCLC) with KRAS mutations exhibit distinct IL-17A-driven inflammatory profiles, which are modulated by glutamine metabolism and mitochondrial ROS, influencing therapeutic responses (46).

Collectively, these intertwined metabolic alterations—encompassing nutrient deprivation, mitochondrial damage, and metabolite-driven signaling—create a profoundly immunosuppressive milieu. Targeting these pathways to restore metabolic fitness in cytotoxic cells, such as with LDHA inhibitors or IL-15 to restore OXPHOS in NK cells, not only underscores metabolic reprogramming as a central mechanism in immune evasion, but also represents a promising therapeutic strategy to reverse immune dysfunction (47).

### 3.3 Underappreciated metabolic roles of stromal cells

Within the TME, stromal cells, particularly CAFs, orchestrate an immunosuppressive landscape through profound metabolic alterations. Firstly, CAFs contribute significantly to a lactate-rich milieu by enhancing glycolytic flux and engaging in metabolic symbiosis with cancer cells (48). Elevated extracellular lactate directly impairs CTL function by disrupting their proliferation, cytokine production, and cytotoxic capacity (49). Secondly, stromal cells prominently express ectonucleotidases like CD39 and CD73, which convert pro-inflammatory extracellular ATP into potent immunosuppressive adenosine (50, 51). This accumulation of adenosine subsequently engages A2A receptors on T cells, effectively curbing their activation and effector functions (50, 52). Furthermore, competition for essential nutrients like glutamine further restricts available resources for infiltrating immune cells. Collectively, these metabolic activities create a hostile environment that fosters immune evasion and presents a significant barrier to the efficacy of cancer therapies. Targeting these stromal-driven metabolic pathways is therefore under active clinical investigation as a strategy to reinvigorate anti-tumor immune responses (53).

## 4 Metabolic regulation of tumor immunotherapies

Tumor immunotherapy is a treatment that recognizes and eliminates tumor cells by activating or enhancing the body’s immune system. In the past decade, breakthrough progress has been made in tumor immunotherapy. Immune checkpoint inhibitor(CTLA-4/PD-1 blockade), CAR-T cell therapy, and therapeutic cancer vaccines have markedly improved survival outcomes in malignancies by either reversing T-cell suppression or enabling tumor-specific cytotoxicity. Emerging strategies targeting immunosuppressive components of the TME and novel bispecific T-cell engagers (BiTEs) have effectively countered tumor immune evasion mechanisms (36). The efficacy of these immunotherapies is intrinsically linked to metabolic regulation within the tumor-immune ecosystem throughout immunometabolic editing process.

### 4.1 Cell type-specific immunometabolic regulation

Researchers emphasize the application of T cell metabolism reprogramming in precision immunotherapy. For instance, the cholesterol esterase ACAT1 is a key regulatory target for T cell metabolism. Inhibition of its activity can enhance the anti-tumor function of CD8^+^ T cells. This is because elevated cholesterol concentrations within the plasma membrane of CD8^+^ T lymphocytes potentiate T-cell receptor (TCR) clustering and signal amplification, concomitant with heightened efficacy in immunological synapse assembly (47). Moreover, mannose metabolism governs CD8^+^ T cell stemness and antitumor efficacy through OGT-mediated β-catenin O-GlcNAcylation, which sustains Tcf7-dependent epigenetic reprogramming to decouple proliferation from exhaustion, while D-mannose supplementation during *in vitro* expansion generates stem-like T cell products with enhanced therapeutic potential for adoptive immunotherapy (54).

Apart from T cell metabolism regulation, researchers explored strategies to enhance tumor immunotherapy through targeted macrophage metabolism, highlighting the role of PHGDH in reversing the TAM immunosuppressive phenotype. The PHGDH-catalyzed *de novo* serine biosynthesis pathway critically regulates the glutaminolysis by converting glutamate to α-KG. This metabolic shift is indispensable for amino acid-dependent mTORC1 signaling activation, thereby sustaining polarization of immunosuppressive M2-like macrophages and TAM expansion (55). Another study revealed the role of the cholesterol metabolizer CH25H and its metabolite 25-HC in inhibiting macrophage activation. 25-HC could promote tumor growth by upregulating the expression of Arg1, Il10, and Mrc1 of macrophages, thus providing a new metabolic target for tumor immunotherapy (56).

### 4.2 Metabolite-governed immunometabolic regulation

Metabolites such as lactate or cholesterol are regarded as potential targets. Reducing lactate production and accumulation by regulating lactic acid metabolism has been found to enhance the efficacy of immunotherapy. Since the expression of PD-L1 is regulated by high lactate levels in the TME, blocking the lactate-generating enzyme LDH-A can enhance the efficacy of anti-PD-1 treatment (57). Moreover, PCSK9 has been found to affect the anti-tumor immune response by regulating the surface LDLR expression of CD8^+^ T cells. Reduced LDLR expression may critically govern intracellular cholesterol homeostasis in CD8^+^ TILs, thereby contributing to dysregulated metabolic states within the TME (58). Researchers also explored the role of N6-methyladenosine RNA methylation as a metabolic reprogramming marker in the immune microenvironment, and proposed new immunotherapies regulating m6A modifications. It was reported that the enrichment of m6A within the 3′-UTR of PD-L1 mRNA, demonstrating that JNK signaling promotes immune escape in bladder cancer via METTL3-mediated m6A modification (59).

## 5 Tumor immunometabolism targeted therapeutic strategies

Therapeutic targeting of cancer metabolic reprogramming represents a paradigm shift in oncology, focusing on vulnerabilities spanning tumor-intrinsic pathways, immune cell modulation and microenvironmental interactions, within the framework of immuonmetabolic editing.

### 5.1 Targeting metabolic pathways

Tumor cells prioritize glycolysis and glutaminolysis to fuel proliferation. Glutamine metabolism, catalyzed by GLS, supports nucleotide synthesis and redox balance. GLS inhibitors like CB-839 disrupt glutamine-to-glutamate conversion, elevating ROS and sensitizing tumors to chemotherapy in pancreatic and ovarian cancers (60). Similarly, glycolysis inhibitors such as 2-deoxyglucose (2-DG) target HK2, inducing ROS-mediated cell apoptosis (61). LDHA inhibitor FX11 suppresses lactate production, triggering oxidative stress and tumor regression in human lymphoma and pancreatic models (62). D-Mannose emerges as a promising modulator, countering T cell exhaustion by restoring metabolic balance. Mechanistically, D-mannose activates AMPK, leading to phosphorylation of PD-L1 at S195, which induces its abnormal glycosylation and proteasomal degradation (63). This degradation enhances T cell activation and cytotoxic killing (64). Preclinically, D-mannose synergizes with PD-1 blockade, significantly inhibiting triple-negative breast cancer (TNBC) growth and increasing CD8^+^ T cell infiltration, offering a strategy to overcome immunotherapy resistance (63). Adenosine accumulation within the TME potently suppresses T and NK cell function via the A2A receptor (A2AR). A2AR antagonists like CPI-444 block adenosine-mediated cAMP production and restore TCR signaling, IL-2, and IFN-γ production in T cells (65). Critically, CPI-444 demonstrates clinical activity as a single agent in renal cell carcinoma (RCC) patients, including those refractory to PD-1/PD-L1 blockade (66). Combining CPI-444 with anti-PD-L1 achieves synergistic tumor elimination in preclinical models, indicating its potential to reverse checkpoint inhibitor resistance (67, 68). Arginine metabolism is hijacked by tumors to “educate” immunosuppressive TAMs. Cancer cell-derived arginine fuels polyamine biosynthes in TAMs (69). Targeting this axis, the ornithine decarboxylase (ODC) inhibitor α-difluoromethylornithine (DFMO) disrupts myeloid-driven immunosuppression. DFMO impairs the suppressive function of MDSCs by reducing arginase activity and inhibiting the CD39/CD73-adenosine pathway (70). Furthermore, DFMO enhances anti-tumor CD8^+^ T cell infiltration and IFN-γ production, demonstrating its role in re-invigorating adaptive immunity (70).

### 5.2 Targeting metabolic enzymes and epigenetic regulators

Metabolic enzymes like pyruvate kinase M2 (PKM2) and GLUTs are pivotal in regulating metabolism in TME. PKM2 activators like TEPP-46 shift glycolysis toward oxidative phosphorylation, depleting biosynthetic intermediates and impeding cell growth (71). GLUT1 inhibitors (WZB117, BAY876) block glucose uptake in HCC and glioblastoma, synergizing with hypoxia-targeting agents (72). Similarly, researchers identified FTO as a demethylase that enhances glycolytic metabolism to evade immune surveillance. They developed Dac51, an FTO-inhibiting compound that disrupts tumor glycolysis and restores CD8^+^ T cell-mediated anti-tumor efficacy (48).

Epigenetic modifiers regulate metabolic genes. For example, HDAC4 deacetylates and activates GLS, promoting lung tumorigenesis, while HDAC inhibitors restore acetyl-CoA levels, normalizing histone acetylation and metabolic gene expression (73). DNMT inhibitors reverse methylation-induced silencing of tumor suppressors like PTEN, ameliorating PI3K/AKT-driven glycolytic flux (74). IDO1 and TDO2 are key immunosuppressive enzymes overexpressed in many cancers. They catalyze tryptophan degradation into kynurenine, depleting local tryptophan and accumulating immunosuppressive metabolites. This activates the GCN2 pathway in T cells and the AhR pathway, which promotes Treg differentiation while inhibiting effector T and NK cells (75). Epacadostat—a selective IDO1 inhibitor binding heme iron—effectively normalized plasma kynurenine levels in phase I trials, but monotherapy showed limited objective responses in solid tumors (76). Conversely, indoximod (a non-competitive IDO1 inhibitor modulating AhR/GCN2) combined with pembrolizumab achieved a 51% objective response rate (ORR) and 70% disease control rate (DCR) in advanced melanoma in a phase II trial. GLS1, the rate-limiting enzyme converting glutamine to glutamate, fuels tumor growth by replenishing TCA cycle intermediates and supporting biosynthesis. CB-839, an oral GLS inhibitor, demonstrated promising combinatorial efficacy: with cabozantinib in metastatic renal cell carcinoma (mRCC), it achieved a 50% ORR and 100% DCR in clear-cell subtypes (77). In myelodysplastic syndrome (MDS), CB-839 plus azacitidine induced marrow complete responses in 62.5% of patients, including those with TP53 mutations/complex karyotypes (78). Sirpiglenastat (DRP-104), a newer GLS inhibitor, is under evaluation with atezolizumab in advanced solid tumors (79). ARG1, expressed by MDSCs and TAMs, depletes arginine in TME, impairing T-cell receptor expression and proliferation. OATD-02, a dual ARG1/2 inhibitor, reverses TME immunosuppression and is undergoing trials in colorectal, renal, and lung cancers (80). Preclinically, L-norvaline (a non-competitive arginase inhibitor) enhances T-cell function and reduces microglial activation in murine models, though it remains investigational (81, 82). We have illustrated more therapeutic targets in **Table 2**.

**Table 2:** An overview of therapeutic targets in tumor immunometabolism.

Category	Drug name/Type	Targeted molecule/Pathway	Mechanism/Action	Clinical indications/Evidence
Targeting Metabolic Pathways	CB-839	GLS	Inhibits glutamine-to-glutamate conversion;Elevates ROS, sensitizes tumors to chemotherapy	Pancreatic/ovarian cancers; Clinical trials show synergy with chemo/immunotherapy
	2-DG	HK2	Glycolysis inhibitor; Induces ROS-mediated apoptosis	Preclinical models (lymphoma, pancreatic)
	FX11	LDHA	Suppresses lactate production; Triggers oxidative stress	Lymphoma/pancreatic models; tumor regression
	D-Mannose	AMPK/PD-L1 axis	Activates AMPK → phosphorylates PD-L1 → induces proteasomal degradation; Enhances T-cell activation	Synergizes with PD-1 blockade in TNBC; Increases CD8^+^ T-cell infiltration; Overcomes immunotherapy resistance
	CPI-444	A2AR	Antagonizes adenosine binding → blocks cAMP production → restores TCR signaling, IL-2/IFN-γ production	Renal cell carcinoma (refractory to PD-1/PD-L1i); Synergizes with anti-PD-L1 in preclinical models
Targeting Metabolic Enzymes and Epigenetic Regulators	TEPP-46	PKM2	PKM2 activator; Shifts glycolysis to oxidative phosphorylation → depletes biosynthetic intermediates	Preclinical tumor growth inhibition
	Romidepsin	HDAC Class I	Promotes histone acetylation; Reduces glucose uptake/glycolysis	Cutaneous T-cell lymphoma, peripheral T-cell lymphoma; ORR 34%, median DoR 15–28 months
	Vorinostat/Panobinostat	HDACs	Induces histone hyperacetylation; Modulates metabolic gene expression	T-cell lymphoma, multiple myeloma
	5-Aza-2’-deoxycytidine (Decitabine)	DNMT	Covalent trapping of DNMT → DNA hypomethylation → reactivates tumor suppressor genes	ORR 30–50% in high-risk MDS/AML; myelosuppression is primary toxicity
	Valproate	HDACs	Inhibits histone deacetylase → increases histone acetylation	Leukemia (combined with decitabine: ORR 22%, CR 19%); migraine prophylaxis

GLS, Glutaminase; 2-DG, 2-Deoxyglucose; HK2, Hexokinase 2; ROS, reactive oxygen species; LDHA, Lactate Dehydrogenase A; A2AR, A2A Receptor; DNMT, DNA Methyltransferase; ORR, Objective Response Rate; DoR, Duration of Response; CR, Complete Response.

### 5.3 Immune checkpoints and metabolic crossroads

PD-1/PD-L1 axis directly impairs CD8^+^ T cell metabolism. PD-1 signaling suppresses glycolysis and mitochondrial biogenesis via PGC-1α inhibition, blunting effector function. Conversely, lactate from tumor cells acidifies TME, upregulating PD-L1 expression and inducing Treg differentiation through MCT1-dependent lactate uptake, thereby dampening anti-tumor immunity (35, 83). Combining PD-1 blockade with LDHA inhibitors (FX11) or MCT1 inhibitors (AZD3965) reverses T cell exhaustion and enhances checkpoint efficacy (61, 62).

### 5.4 Tumor microenvironment modulation

HIF-1α upregulates GLUT1, LDHA, and CAIX, exacerbating glycolysis and acidification. HIF inhibitors normalize vascularization and glucose metabolism (84). CAIX inhibitors (e.g., SLC-0111) buffer pH and restore T cell cytotoxicity in cancer (85). Tumor cells outcompete T cells for glucose and glutamine. Glutamine blockade (CB-839) or adenosine receptor antagonists reverse CD8^+^ T cell suppression (60, 86). CAFs fuel tumor growth via “lactate shuttle” (MCT4-mediated export), while MCT4 inhibition disrupts this metabolic symbiosis (87).

## 6 Technological frontiers in dissecting tumor immunometabolism

Recent advancements in tumor immunology have shifted the paradigm from merely targeting immune checkpoints to a deeper interrogation of the metabolic cross-talk between cancer cells and the immune microenvironment. Single-cell multi-omics technologies have emerged as a cornerstone, enabling the dissection of cellular heterogeneity at unprecedented resolution. By integrating transcriptomic, proteomic, and spatial data, researchers can now map the intricate landscape of immune infiltration and identify specific metabolic signatures—such as cholesterol or branched-chain amino acid (BCAA) profiles—that correlate with therapeutic response (88, 89). This has led to the identification of actionable targets like *DHCR7* and *HMGCS1*, demonstrating that metabolic reprogramming is not just a bystander effect but a critical driver of immune evasion.

Complementing these genomic approaches, hyperpolarized magnetic resonance imaging (HP-MRI) has revolutionized non-invasive metabolic phenotyping. By detecting real-time fluxes of labeled pyruvate into lactate, this technique allows clinicians to visualize tumor heterogeneity and monitor treatment-induced metabolic shifts without tissue destruction (90, 91). Furthermore, the integration of CRISPR-Cas9 screening with immunotherapy has unlocked new pathways; for instance, targeting enzymes involved in ferroptosis or nucleotide metabolism has been shown to sensitize tumors to immune checkpoint blockade, highlighting the potential of genetic engineering to overcome resistance mechanisms (92, 93). Finally, the development of metabolism-regulating nanocarriers represents a cutting-edge delivery strategy. These platforms exploit the TME to release drugs that simultaneously disrupt metabolic homeostasis and trigger immunogenic cell death, achieving spatiotemporal orchestration of therapy (94, 95).

## 7 Concluding remarks and challenges

Metabolic reprogramming extends beyond the intrinsic alterations within tumor cells to profoundly reshape the metabolic landscape of TME. The immunometabolic editing landscape orchestrates microenvironmental immunosuppression by directly subverting the metabolic pathways essential for effector immune cells while concurrently fueling the activity and function of immunosuppressive populations (23, 96). This review synthesizes these advances to elucidate mechanistic links between immunometabolic editing and cancer pathology, offering a roadmap for next-generation therapeutic combinations.

The field of tumor immunometabolism still faces significant hurdles in clinical translation. The spatiotemporal metabolic heterogeneity within the TME, where divergent metabolic demands exist among immune cell subsets and tumor cells. This complexity renders single-target interventions insufficient; spatial metabolomics and real-time monitoring are needed to address dynamic shifts like hypoxia and nutrient competition (5). Balancing metabolic intervention toxicity remains critical, as targeting core pathways risks systemic toxicity in normal cells, necessitating precision drug design (97). The crosstalk between metabolites and immune signaling is inadequately mapped: metabolites like fumarate and PAGln modulate immune receptors through unclear multi-target mechanisms, requiring integrated multi-omics approaches (98). Additionally, synergistic mechanisms between metabolic checkpoints (IDO, GPR34) and classical immune checkpoints (PD-1) need more in-depth exploration for effective combination therapies (23). Clinical translation bottlenecks is another intractable issue, which exists in most strategies in preclinical stages. Reliable biomarkers (e.g., ADSL phosphorylation for STING response) lack large-scale validation (5). Pharmacokinetic limitations due to poor TME drug penetration and compensatory metabolic pathway activation further hinder the efficacy of anti-tumor therapies (99). Emerging tools like tumor-on-chip platforms and spatial metabolomics offer promise for resolving TME dynamics (100), but interdisciplinary efforts are still essential to overcome these barriers.

## References

[1] DeBerardinis RJ Chandel NS . Fundamentals of cancer metabolism. Sci Adv. (2016) 2:e1600200. doi: 10.1126/sciadv.1600200, PMID: 27386546 PMC4928883

[2] Faubert B Solmonson A DeBerardinis RJ . Metabolic reprogramming and cancer progression. Science. (2020) 368:. doi: 10.1126/science.aaw5473, PMID: 32273439 PMC7227780

[3] Xiong J Fu Y Huang J Wang Y Jin X Wan X . Metabolic and senescence characteristics associated with the immune microenvironment in ovarian cancer. Front Endocrinol (Lausanne). (2023) 14:1265525. doi: 10.3389/fendo.2023.1265525, PMID: 38075052 PMC10702973

[4] Ma G Li C Zhang Z Liang Y Liang Z Chen Y . Targeted glucose or glutamine metabolic therapy combined with PD-1/PD-L1 checkpoint blockade immunotherapy for the treatment of tumors - mechanisms and strategies. Front Oncol. (2021) 11:697894. doi: 10.3389/fonc.2021.697894, PMID: 34327138 PMC8314994

[5] Dang Q Li B Jin B Ye Z Lou X Wang T . Cancer immunometabolism: advent, challenges, and perspective. Mol cancer. (2024) 23:72. doi: 10.1186/s12943-024-01981-5, PMID: 38581001 PMC10996263

[6] Vander Heiden MG Cantley LC Thompson CB . Understanding the warburg effect: the metabolic requirements of cell proliferation. Science. (2009) 324:1029–33. doi: 10.1126/science.1160809, PMID: 19460998 PMC2849637

[7] Huang R Lu X Sun X Wu H . Metabolomic profiling of childhood medulloblastoma: contributions and relevance to diagnosis and molecular subtyping. J Cancer Res Clin Oncol. (2024) 150:471. doi: 10.1007/s00432-024-05990-1, PMID: 39441459 PMC11499513

[8] Pavlides S Diana W-M Remedios C-C Neal F WA K FP G . The reverse Warburg effect: Aerobic glycolysis in cancer associated fibroblasts and the tumor stroma. Cell Cycle. (2009) 8:3984–4001. doi: 10.4161/cc.8.23.10238, PMID: 19923890

[9] Jaworska M Szczudło J Pietrzyk A Shah J Trojan SE Ostrowska B . The Warburg effect: a score for many instruments in the concert of cancer and cancer niche cells. Pharmacol Rep. (2023) 75:876–90. doi: 10.1007/s43440-023-00504-1, PMID: 37332080 PMC10374743

[10] Brogna MR Varone V DelSesto M Ferrara G . The role of CAFs in therapeutic resistance in triple negative breast cancer: an emerging challenge. Front Mol Biosci. (2025) 12:1568865. doi: 10.3389/fmolb.2025.1568865, PMID: 40230452 PMC11994926

[11] Elia I Haigis MC . Metabolites and the tumour microenvironment: from cellular mechanisms to systemic metabolism. Nat Metab. (2021) 3:21–32. doi: 10.1038/s42255-020-00317-z, PMID: 33398194 PMC8097259

[12] Chen C Wang Z Ding Y Qin Y . Tumor microenvironment-mediated immune evasion in hepatocellular carcinoma. Front Immunol. (2023) 14:1133308. doi: 10.3389/fimmu.2023.1133308, PMID: 36845131 PMC9950271

[13] Li X Yang Y Zhang B Lin X Fu X An Y . Lactate metabolism in human health and disease. Signal transduction targeted Ther. (2022) 7:305. doi: 10.1038/s41392-022-01151-3, PMID: 36050306 PMC9434547

[14] Lan Z Lv S Ge Z Zhao B Li L Li C . Lactic acid regulates lipid droplet aggregation through a microglia-neuron axis in neuroinflammation. J Lipid Res. (2024) 65. doi: 10.1016/j.jlr.2024.100629, PMID: 39182605 PMC11437955

[15] Bullock TN Dong H . Metabolic influences that regulate dendritic cell function in tumors. Front Immunol. (2014) 5:24. doi: 10.3389/fimmu.2014.00024, PMID: 24523723 PMC3906600

[16] Semenza GL . HIF-1 mediates metabolic responses to intratumoral hypoxia and oncogenic mutations. J Clin Invest. (2013) 123:3664–71. doi: 10.1172/JCI67230, PMID: 23999440 PMC3754249

[17] Su P Wang Q Bi E Ma X Liu L Yang M . Enhanced lipid accumulation and metabolism are required for the differentiation and activation of tumor-associated macrophages. Cancer Res. (2020) 80:1438–50. doi: 10.1158/0008-5472.CAN-19-2994, PMID: 32015091 PMC7127942

[18] Martin-Perez M Urdiroz-Urricelqui U Bigas C Benitah SA . The role of lipids in cancer progression and metastasis. Cell Metab. (2022) 34:1675–99. doi: 10.1016/j.cmet.2022.09.023, PMID: 36261043

[19] Luo Q Zheng N Jiang L Wang T Zhang P Liu Y . Lipid accumulation in macrophages confers protumorigenic polarization and immunity in gastric cancer. Cancer Sci. (2020) 111:4000–11. doi: 10.1111/cas.14616, PMID: 32798273 PMC7648032

[20] Li Z Liu H Luo X . Lipid droplet and its implication in cancer progression. American journal of cancer research (2020) 10 12:4112–22. PMC7783747 33414989

[21] Miranda-Galvis M Teng Y . Targeting hypoxia-driven metabolic reprogramming to constrain tumor progression and metastasis. International Journal of Molecular Sciences. (2020) 21:5487. doi: 10.3390/ijms21155487, PMID: 32751958 PMC7432774

[22] Vasseur S Guillaumond F . Lipids in cancer: a global view of the contribution of lipid pathways to metastatic formation and treatment resistance. Oncogenesis. (2022) 11:46. doi: 10.1038/s41389-022-00420-8, PMID: 35945203 PMC9363460

[23] Xia L Oyang L Lin J Tan S Han Y Wu N . The cancer metabolic reprogramming and immune response. Mol Cancer. (2021) 20:28. 33546704 10.1186/s12943-021-01316-8 PMC7863491

[24] Renner K Singer K Koehl GE Geissler EK Peter K Siska PJ . Metabolic hallmarks of tumor and immune cells in the tumor microenvironment. Front Immunol. (2017) 8:248. doi: 10.3389/fimmu.2017.00248, PMID: 28337200 PMC5340776

[25] Zhang J Shi Z Xu X Yu Z Mi J . The influence of microenvironment on tumor immunotherapy. FEBS J. (2019) 286:4160–75. doi: 10.1111/febs.15028, PMID: 31365790 PMC6899673

[26] Zhou Z Zheng J Lu Y Mai Z Lin Y Lin P . Optimizing CD8(+) T cell-based immunotherapy via metabolic interventions: a comprehensive review of intrinsic and extrinsic modulators. Exp Hematol Oncol. (2024) 13:103. doi: 10.1186/s40164-024-00575-7, PMID: 39438986 PMC11495118

[27] Ma G Zhang Z Li P Zhang Z Zeng M Liang Z . Reprogramming of glutamine metabolism and its impact on immune response in the tumor microenvironment. Cell Communication Signaling. (2022) 20:114. doi: 10.1186/s12964-022-00909-0, PMID: 35897036 PMC9327201

[28] Erick TK Brossay L . Phenotype and functions of conventional and non-conventional NK cells. Curr Opin Immunol. (2016) 38:67–74. doi: 10.1016/j.coi.2015.11.007, PMID: 26706497 PMC4715908

[29] Wang Y Huang R Wang Z Xiong J Wang X Zhang X . Facing challenges with hope: universal immune cells for hematologic Malignancies. Cancer Biol Med. (2023) 20:229–47. doi: 10.20892/j.issn.2095-3941.2022.0759, PMID: 37144558 PMC10157807

[30] Carnevale S Ghasemi S Rigatelli A Jaillon S . The complexity of neutrophils in health and disease: Focus on cancer. Semin Immunol. (2020) 48:101409. doi: 10.1016/j.smim.2020.101409, PMID: 32958359 PMC7500440

[31] Burgos RA Werling D Hermosilla CR . Editorial: The emerging role of metabolism and metabolic-related receptors on neutrophil extracellular traps (NET) formation. Front Immunol. (2022) 13:. doi: 10.3389/fimmu.2022.1028228, PMID: 36238307 PMC9552222

[32] Kumar V Stewart JH . Immunometabolic reprogramming, another cancer hallmark. Front Immunol. (2023) 14:1125874. doi: 10.3389/fimmu.2023.1125874, PMID: 37275901 PMC10235624

[33] Keating ST El-Osta A . Metaboloepigenetics in cancer, immunity, and cardiovascular disease. Cardiovasc Res. (2023) 119:357–70. doi: 10.1093/cvr/cvac058, PMID: 35389425 PMC10064843

[34] Long Y Shi H He Y Qi X . Analyzing the impact of metabolism on immune cells in tumor microenvironment to promote the development of immunotherapy. Front Immunol. (2024) 14:1307228. doi: 10.3389/fimmu.2023.1307228, PMID: 38264667 PMC10804850

[35] Qin S Xie B Wang Q Yang R Sun J Hu C . New insights into immune cells in cancer immunotherapy: from epigenetic modification, metabolic modulation to cell communication. MedComm. (2024) 5:e551. doi: 10.1002/mco2.551, PMID: 38783893 PMC11112485

[36] Emens LA Romero PJ Anderson AC Bruno TC Capitini CM Collyar D . Challenges and opportunities in cancer immunotherapy: a Society for Immunotherapy of Cancer (SITC) strategic vision. J immunotherapy Cancer. (2024) 12(6):e009063. doi: 10.1136/jitc-2024-009063, PMID: 38901879 PMC11191773

[37] Hu C Xuan Y Zhang X Liu Y Yang S Yang K . Immune cell metabolism and metabolic reprogramming. Mol Biol Rep. (2022) 49:9783–95. doi: 10.1007/s11033-022-07474-2, PMID: 35696048 PMC9189272

[38] Palmer CS Ostrowski M Balderson B Christian N Crowe SM . Glucose metabolism regulates T cell activation, differentiation, and functions. Front Immunol. (2015) 6:1. doi: 10.3389/fimmu.2015.00001, PMID: 25657648 PMC4302982

[39] Wang B Pei J Xu S Liu J Yu J . A glutamine tug-of-war between cancer and immune cells: recent advances in unraveling the ongoing battle. J Exp Clin Cancer Res. (2024) 43:74. doi: 10.1186/s13046-024-02994-0, PMID: 38459595 PMC10921613

[40] Yan X Liu C . The ATF4-glutamine axis: a central node in cancer metabolism, stress adaptation, and therapeutic targeting. Cell Death Discovery. (2025) 11:390. doi: 10.1038/s41420-025-02683-7, PMID: 40830338 PMC12365302

[41] Yu Y Liang Y Li D Wang L Liang Z Chen Y . Glucose metabolism involved in PD-L1-mediated immune escape in the Malignant kidney tumour microenvironment. Cell Death Discovery. (2021) 7:15. doi: 10.1038/s41420-021-00401-7, PMID: 33462221 PMC7814120

[42] Liu Z Ning F Cai Y Sheng H Zheng R Yin X . The EGFR-P38 MAPK axis up-regulates PD-L1 through miR-675-5p and down-regulates HLA-ABC via hexokinase-2 in hepatocellular carcinoma cells. Cancer Commun (Lond). (2021) 41:62–78. doi: 10.1002/cac2.12117, PMID: 34236149 PMC7819566

[43] Kim SW Woo KC Young-Ah M Kim HS . Reprogramming of tumor-associated macrophages by metabolites generated from tumor microenvironment. Anim Cells Systems. (2024) 28:123–36. doi: 10.1080/19768354.2024.2336249, PMID: 38577621 PMC10993762

[44] Fischer K Hoffmann P Voelkl S Meidenbauer N Ammer J Edinger M . Inhibitory effect of tumor cell–derived lactic acid on human T cells. Blood. (2007) 109:3812–9. doi: 10.1182/blood-2006-07-035972, PMID: 17255361

[45] Wu L-Y Park S-H Jakobsson H Shackleton M Möller A . Immune regulation and immune therapy in melanoma: review with emphasis on CD155 signalling. Cancers (Basel). (2024) 16:1950. doi: 10.3390/cancers16111950, PMID: 38893071 PMC11171058

[46] Armstrong D Chang C-Y Lazarus DR Corry D Kheradmand F . Lung cancer heterogeneity in modulation of th17/IL17A responses. Frontiers in Oncology. (2019) 9:1384. doi: 10.3389/fonc.2019.01384, PMID: 31921642 PMC6914699

[47] Yang W Bai Y Xiong Y Zhang J Chen S Zheng X . Potentiating the antitumour response of CD8+ T cells by modulating cholesterol metabolism. Nature. (2016) 531:651–5. doi: 10.1038/nature17412, PMID: 26982734 PMC4851431

[48] Kitamura F Semba T Yasuda-Yoshihara N Yamada K Nishimura A Yamasaki J . Cancer-associated fibroblasts reuse cancer-derived lactate to maintain a fibrotic and immunosuppressive microenvironment in pancreatic cancer. JCI Insight. (2023) 8(20):e163022. doi: 10.1172/jci.insight.163022, PMID: 37733442 PMC10619496

[49] Xu Y Hao X Ren Y Xu Q Liu X Song S . Research progress of abnormal lactate metabolism and lactate modification in immunotherapy of hepatocellular carcinoma. Front Oncol. (2023) 12:1063423. doi: 10.3389/fonc.2022.1063423, PMID: 36686771 PMC9853001

[50] Xia C Yin S To KKW Fu L . CD39/CD73/A2AR pathway and cancer immunotherapy. Mol Cancer. (2023) 22:44. doi: 10.1186/s12943-023-01733-x, PMID: 36859386 PMC9979453

[51] Goueli SA Hsiao K . Monitoring and characterizing soluble and membrane-bound ectonucleotidases CD73 and CD39. PloS One. (2019) 14:e0220094. doi: 10.1371/journal.pone.0220094, PMID: 31652269 PMC6814236

[52] de Lourdes Mora-García M García-Rocha R Morales-Ramírez O Montesinos JJ Weiss-Steider B Hernández-Montes J . Mesenchymal stromal cells derived from cervical cancer produce high amounts of adenosine to suppress cytotoxic T lymphocyte functions. J Trans Med. (2016) 14:302. doi: 10.1186/s12967-016-1057-8, PMID: 27782859 PMC5080842

[53] Kaplinsky N Williams K Watkins D Adams M Stanbery L Nemunaitis J . Regulatory role of CD39 and CD73 in tumor immunity. Future Oncol. (2024) 20:1367–80. doi: 10.2217/fon-2023-0871, PMID: 38652041 PMC11321403

[54] Qiu Y Su Y Xie E Cheng H Du J Xu Y . Mannose metabolism reshapes T cell differentiation to enhance anti-tumor immunity. Cancer Cell. (2025) 43:103–21.e8. doi: 10.1016/j.ccell.2024.11.003, PMID: 39642888 PMC11756673

[55] Liu J Ma Z Jia W Lan P . Targeting macrophage metabolism to enhance tumor immunotherapy. Cell Mol Immunol. (2024) 21:530–2. doi: 10.1038/s41423-024-01149-7, PMID: 38632383 PMC11061165

[56] Liu S Wu J Tong X Huang L-H . A novel target to turn cold tumors into hot tumors: lysosomal 25-hydroxycholesterol activates AMPKα and immunosuppressive tumor-associated macrophages. Cell Mol Immunol. (2024) 21:801–3. doi: 10.1038/s41423-024-01171-9, PMID: 38740924 PMC11291875

[57] Daneshmandi S Wegiel B Seth P . Blockade of lactate dehydrogenase-A (LDH-A) improves efficacy of anti-programmed cell death-1 (PD-1) therapy in melanoma. Cancers. (2019) 11(4):450. doi: 10.3390/cancers11040450, PMID: 30934955 PMC6521327

[58] Yuan J Cai T Zheng X Ren Y Qi J Lu X . Potentiating CD8+ T cell antitumor activity by inhibiting PCSK9 to promote LDLR-mediated TCR recycling and signaling. Protein Cell. (2021) 12:240–60. doi: 10.1007/s13238-021-00821-2, PMID: 33606190 PMC8018994

[59] Ni Z Sun P Zheng J Wu M Yang C Cheng M . JNK Signaling Promotes Bladder Cancer Immune Escape by Regulating METTL3-Mediated m6A Modification of PD-L1 mRNA. Cancer Res. (2022) 82:1789–802. doi: 10.1158/0008-5472.CAN-21-1323, PMID: 35502544

[60] Wang Z Liu F Fan N Zhou C Li D Macvicar T . Targeting glutaminolysis: new perspectives to understand cancer development and novel strategies for potential target therapies. Front Oncol. (2020) 10:589508. doi: 10.3389/fonc.2020.589508, PMID: 33194749 PMC7649373

[61] Park JH Pyun WY Park HW . Cancer metabolism: phenotype. Signaling Ther Targets. (2020) 9:2308. doi: 10.3390/cells9102308, PMID: 33081387 PMC7602974

[62] Le A Cooper CR Gouw AM Dinavahi R Maitra A Deck LM . Inhibition of lactate dehydrogenase A induces oxidative stress and inhibits tumor progression. Proc Natl Acad Sci U S A. (2010) 107:2037–42. doi: 10.1073/pnas.0914433107, PMID: 20133848 PMC2836706

[63] Zhang R Yang Y Dong W Lin M He J Zhang X . D-mannose facilitates immunotherapy and radiotherapy of triple-negative breast cancer via degradation of PD-L1. Proc Natl Acad Sci U S A. (2022) 119:e2114851119. doi: 10.1073/pnas.2114851119, PMID: 35181605 PMC8872783

[64] Zhai H Zhang N Mo D Qin T . CCL20 is a potential therapeutic target associated with immune infiltration in breast cancer. J Int Med Res. (2023) 51(8):3000605231171762. doi: 10.1177/03000605231171762, PMID: 37571985 PMC10423453

[65] Willingham SB Ho PY Hotson A Hill C Piccione EC Hsieh J . A2AR antagonism with CPI-444 induces antitumor responses and augments efficacy to anti–PD-(L)1 and anti–CTLA-4 in preclinical models. Cancer Immunol Res. (2018) 6:1136–49. doi: 10.1158/2326-6066.CIR-18-0056, PMID: 30131376

[66] Fong L Forde PM Powderly JD Goldman JW Nemunaitis JJ Luke JJ . Safety and clinical activity of adenosine A2a receptor (A2aR) antagonist, CPI-444, in anti-PD1/PDL1 treatment-refractory renal cell (RCC) and non-small cell lung cancer (NSCLC) patients. J Clin Oncol (2017) 35:3004. doi: 10.1200/JCO.2017.35.15_suppl.3004

[67] Willingham S Hotson A Ho P Choy C Walter K Yusko E . Abstract 5593: Inhibition of A2AR induces anti-tumor immunity alone and in combination with anti-PD-L1 in preclinical and clinical studies. Cancer Res. (2017) 77:5593. doi: 10.1158/1538-7445.AM2017-5593

[68] Wang L Zhang J Zhang W Zheng M Guo H Pan X . The inhibitory effect of adenosine on tumor adaptive immunity and intervention strategies. Acta Pharm Sin B. (2024) 14:1951–64. doi: 10.1016/j.apsb.2023.12.004, PMID: 38799637 PMC11119508

[69] Wang J Deng S Cheng D Gu J Qin L Mao F . Engineered microparticles modulate arginine metabolism to repolarize tumor-associated macrophages for refractory colorectal cancer treatment. J Trans Med. (2024) 22:908. doi: 10.1186/s12967-024-05652-3, PMID: 39375706 PMC11457421

[70] Ye C Geng Z Dominguez D Chen S Fan J Qin L . Targeting ornithine decarboxylase by α-difluoromethylornithine inhibits tumor growth by impairing myeloid-derived suppressor cells. J Immunol. (2016) 196:915–23. doi: 10.4049/jimmunol.1500729, PMID: 26663722 PMC4707077

[71] Li C Zhang G Zhao L Ma Z Chen H . Metabolic reprogramming in cancer cells: glycolysis, glutaminolysis, and Bcl-2 proteins as novel therapeutic targets for cancer. World J Surg Oncol. (2016) 14:15. doi: 10.1186/s12957-016-0769-9, PMID: 26791262 PMC4721116

[72] Zhang Y Li Q Huang Z Li B Nice EC Huang C . Targeting glucose metabolism enzymes in cancer treatment: current and emerging strategies. Cancers. (2022) 14:4568. doi: 10.3390/cancers14194568, PMID: 36230492 PMC9559313

[73] Wang T Lu Z Han T Wang Y Gan M Wang J-B . Deacetylation of glutaminase by HDAC4 contributes to lung cancer tumorigenesis. Int J Biol Sci. (2022) 18:4452–65. doi: 10.7150/ijbs.69882, PMID: 35864951 PMC9295053

[74] Liu Z Ren Y Weng S Xu H Li L Han X . A new trend in cancer treatment: the combination of epigenetics and immunotherapy. Front Immunol. (2022) 13:809761. doi: 10.3389/fimmu.2022.809761, PMID: 35140720 PMC8818678

[75] Grobben Y . Targeting amino acid-metabolizing enzymes for cancer immunotherapy. Front Immunol. (2024) 15:1440269. doi: 10.3389/fimmu.2024.1440269, PMID: 39211039 PMC11359565

[76] Beatty GL O’Dwyer PJ Clark J Shi JG Bowman KJ Scherle PA . First-in-human phase I study of the oral inhibitor of indoleamine 2,3-dioxygenase-1 epacadostat (INCB024360) in patients with advanced solid Malignancies. Clin Cancer Res. (2017) 23:3269–76. doi: 10.1158/1078-0432.CCR-16-2272, PMID: 28053021 PMC5496788

[77] Meric-Bernstam F Lee RJ Carthon BC Iliopoulos O Mier JW Patel MR . CB-839, a glutaminase inhibitor, in combination with cabozantinib in patients with clear cell and papillary metastatic renal cell cancer (mRCC): Results of a phase I study. J Clin Oncol (2019) 37:549. doi: 10.1200/JCO.2019.37.7_suppl.549

[78] Guerra V Dinardo CD Konopleva M Burger JA Borthakur G Jabbour E . Interim results from a phase Ib/II clinical study of the glutaminase inhibitor telaglenastat (CB-839) in combination with azacitidine in patients with advanced myelodysplastic syndrome (MDS). J Clin Oncol (2019) 37:7037. doi: 10.1200/JCO.2019.37.15_suppl.7037

[79] Ni R Li Z Li L Peng D Ming Y Li L . Rethinking glutamine metabolism and the regulation of glutamine addiction by oncogenes in cancer. Front Oncol. (2023) 13:1143798. doi: 10.3389/fonc.2023.1143798, PMID: 36959802 PMC10029103

[80] Susanne MS Mark KB Jason C Ethan E Tony H Julie RJ . Inhibition of arginase by CB-1158 blocks myeloid cell-mediated immune suppression in the tumor microenvironment. J immunotherapy cancer. (2017) 5:101. doi: 10.1186/s40425-017-0308-4, PMID: 29254508 PMC5735564

[81] Dobhal S Baliyan S Singh MF Bisht S Setya S . Amelioration of the abnormalities associated with metabolic syndrome by L-norvaline in hyperlipidemic diabetic rats. Eur Pharm J. (2022) 68:16–26. doi: 10.2478/afpuc-2021-0015

[82] Polis B Srikanth KD Elliott E Gil-Henn H Samson AO . L-norvaline reverses cognitive decline and synaptic loss in a murine model of alzheimer’s disease. Neurotherapeutics. (2018) 15:1036–54. doi: 10.1007/s13311-018-0669-5, PMID: 30288668 PMC6277292

[83] Li X Zhou L Xu X Liu X Wu W Feng Q . Metabolic reprogramming in hepatocellular carcinoma: a bibliometric and visualized study from 2011 to 2023. Front Pharmacol. (2024) 15:1392241. doi: 10.3389/fphar.2024.1392241, PMID: 39086383 PMC11289777

[84] Magar AG Morya VK Kwak MK Oh JU Noh KC . A molecular perspective on HIF-1α and angiogenic stimulator networks and their role in solid tumors: an update. Int J Mol Sci. (2024) 25:3313. doi: 10.3390/ijms25063313, PMID: 38542288 PMC10970012

[85] Phan LM Yeung SC Lee MH . Cancer metabolic reprogramming: importance, main features, and potentials for precise targeted anti-cancer therapies. Cancer Biol Med. (2014) 11:1–19. doi: 10.7497/j.issn.2095-3941.2014.01.001, PMID: 24738035 PMC3969803

[86] Wang X Zhou L Wang H Chen W Jiang L Ming G . Metabolic reprogramming, autophagy, and ferroptosis: Novel arsenals to overcome immunotherapy resistance in gastrointestinal cancer. Cancer Med. (2023) 12:20573–89. doi: 10.1002/cam4.6623, PMID: 37860928 PMC10660574

[87] Gupta S Roy A Dwarakanath BS . Metabolic cooperation and competition in the tumor microenvironment: implications for therapy. Front Oncol. (2017) 7:68. doi: 10.3389/fonc.2017.00068, PMID: 28447025 PMC5388702

[88] Le J Dian Y Zhao D Guo Z Luo Z Chen X . Single-cell multi-omics in cancer immunotherapy: from tumor heterogeneity to personalized precision treatment. Mol Cancer. (2025) 24:221. doi: 10.1186/s12943-025-02426-3, PMID: 40855431 PMC12376342

[89] Du M Zheng J Zhou G Zhuang Y Huang C Ye W . Cholesterol metabolism shapes immune low-response states in LUAD: a multi-omics cholesterol metabolism signature predicts immunotherapy benefit and identifies DHCR7 as a therapeutic target. Front Immunol. (2025) 16:. doi: 10.3389/fimmu.2025.1696360, PMID: 41246332 PMC12611822

[90] Guglielmetti C Cordano C Najac C Green AJ Chaumeil MM . Imaging immunomodulatory treatment responses in a multiple sclerosis mouse model using hyperpolarized 13C metabolic MRI. Commun Med. (2023) 3:71. doi: 10.1038/s43856-023-00300-1, PMID: 37217574 PMC10202949

[91] Dou Q Grant AK Coutinto de Souza P Moussa M Nasser I Ahmed M . Characterizing metabolic heterogeneity of hepatocellular carcinoma with hyperpolarized 13C pyruvate MRI and mass spectrometry. Radiol Imaging Cancer. (2024) 6:. doi: 10.1148/rycan.230056, PMID: 38426887 PMC10988335

[92] Yao F Zhou S Zhang R Chen Y Huang W Yu K . CRISPR/Cas9 screen reveals that targeting TRIM34 enhances ferroptosis sensitivity and augments immunotherapy efficacy in hepatocellular carcinoma. Cancer Letters. (2024) 593:216935. doi: 10.1016/j.canlet.2024.216935, PMID: 38704136

[93] Li Y-R Lyu Z Tian Y Fang Y Zhu Y Chen Y . Advancements in CRISPR screens for the development of cancer immunotherapy strategies. Mol Ther - Oncolytics. (2023) 31. doi: 10.1016/j.omto.2023.100733, PMID: 37876793 PMC10591018

[94] Guo Y Li Y Zhang M Ma R Wang Y Weng X . Polymeric nanocarrier via metabolism regulation mediates immunogenic cell death with spatiotemporal orchestration for cancer immunotherapy. Nat Commun. (2024) 15:8586. doi: 10.1038/s41467-024-53010-0, PMID: 39362879 PMC11450208

[95] Zhang F Cheng K Zhang X-S Zhou S Zou J-H Tian M-Y . Cascade-catalysed nanocarrier degradation for regulating metabolism homeostasis and enhancing drug penetration on breast cancer. J Nanobiotechnology. (2024) 22:680. doi: 10.1186/s12951-024-02948-w, PMID: 39506777 PMC11542379

[96] Cognet G Muir A . Identifying metabolic limitations in the tumor microenvironment. Sci Adv. (2024) 10:eadq7305. doi: 10.1126/sciadv.adq7305, PMID: 39356752 PMC11446263

[97] Ohe T Takahashi K Nakamura S Mashino T . Strategic drug design to avoid the metabolic activation of hepatotoxic drugs. Yakugaku zasshi: J Pharm Soc Japan. (2017) 137:249–55. doi: 10.1248/yakushi.16-00230-1, PMID: 28250317

[98] Aderinto N Abdulbasit MO Tangmi ADE Okesanya JO Mubarak JM . Unveiling the growing significance of metabolism in modulating immune cell function: exploring mechanisms and implications; a review. Ann Med Surg (2012). (2023) 85:5511–22. doi: 10.1097/MS9.0000000000001308, PMID: 37915697 PMC10617839

[99] Li Y Meng Q Yang M Liu D Hou X Tang L . Current trends in drug metabolism and pharmacokinetics. Acta Pharm Sin B. (2019) 9:1113–44. doi: 10.1016/j.apsb.2019.10.001, PMID: 31867160 PMC6900561

[100] Xu S Li X Ma W . Redefining the tumor microenvironment with emerging therapeutic strategies. Oncol Res. (2024) 32:1701–8. 10.32604/or.2024.055161 PMC11497178 39449800

